# Acacetin resists UVA photoaging by mediating the SIRT3/ROS/MAPKs pathway

**DOI:** 10.1111/jcmm.17415

**Published:** 2022-06-28

**Authors:** Jing Mu, Hong Chen, Mengyi Ye, Xiaoxia Zhang, Huisheng Ma

**Affiliations:** ^1^ School of Traditional Chinese Medicine Ningxia Medical University Yinchuan China

**Keywords:** acacetin, MAPK, ROS, SIRT3, UVA irradiation

## Abstract

Ultraviolet A (UVA) radiation is a major contributor to the pathogenesis of skin photoaging, and the aim of this study was to investigate the effect of Acacetin on skin photoaging in UVA‐irradiated mice and human dermal fibroblasts (HDF). Healthy dorsal depilated rats were irradiated with UVA 30 J/cm^2^ daily, every other day, for 1 month. Acacetin (40, 80 mg kg/day) was coated to the bare skin of the rats' backs 1 h before UVA irradiation. HDF were treated different concentrations of Acacetin (5, 10, 20 μg/ml) and then irradiated with UVA (20 J/cm^2^). Acacetin was found to be effective in ameliorating UVA‐induced oxidative stress and cell death. Acacetin also prevented the UVA‐induced decrease of SIRT3, reduced the activation of mitogen‐activated protein kinases (MAPKs, p‐38 and p‐JNK) and blocked the down‐regulated activation of oxidative stress in matrix metalloproteinases (MMPs). In addition, Acacetin increased the expressions of collagen‐promoting proteins (TGF‐β and Smad3). Finally, the SIRT3 inhibitor 3‐TYP blocked all protective effects of Acacetin, indicating that the protective effect of Acacetin against UVA photoaging is SIRT3‐dependent. Acacetin effectively mitigated photoaging by targeting the promotion of SIRT3, inhibiting the UVA‐induced increases in MMPs and pro‐inflammatory factors, and promoting TGF‐β and Smad3.

## Graphical abstract

Acacetin prevents UVA‐induced photoaging by decreasing oxidative stress levels and increasing SIRT3 expression. Acacetin effectively alleviated the photoaging process by inhibiting UVA‐induced MMPs expressions and suppressing the increase of pro‐inflammatory factors, promoting TGF‐β and Smad3.

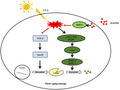

## 1 INTRODUCTION

Among the different wavelengths of solar radiation, ultraviolet (UV) radiation is considered to be the cause of skin cancer and aging.^1^ Of these, UVA (320–400 nm) is considered to be the main cause of skin photoaging, as it penetrates deeper into the dermis layer of the skin and damages elastin and collagen fibres.^2^ SIRT3 is known to promote the scavenging of mitochondrial ROS by participating in the direct deacetylation of SOD2 and is emerging as a key oxidative stress regulator. Previous study has shown that UVA exposure increases the expression of ROS, which activates mitogen‐activated protein kinases (MAPK), upregulates the expressions of c‐Fos and c‐Jun, and induces AP‐1 to regulate the activation of MMP‐1.^3^ Reactive oxygen species (ROS) are associated with the activation of matrix metalloproteinases (MMPs), which have been shown to be associated with collagen degradation in the skin, leading to sagging and wrinkling of the skin.^4^The degradation of collagen I is triggered by MMP‐1 and the process is sustained by MMP‐3, which ultimately impairs dermal function.^5^ Collagen I synthesis is influenced by the activity of transforming growth factor‐β (TGF‐β).^6^ In addition, Smad proteins act as transcription factors to increase collagen synthesis.

Acacetin (5,7‐dihydroxy‐4′‐methoxyflavone) is a natural flavonoid derived from Acacia farnesiana (Linn.) Willd. with various pharmacological activities, such as anti‐cancer, anti‐inflammation and anti‐aging.^7^ In recent study, Acacetin can regulate the expression of collagen I by modulating the TGF‐β/Smad3 signalling pathway.^8^ However, the role of Acacetin in regulating photoaging damage remains to be elucidated. In the present study, Acacetin (A860582, purity ≥98%) was purchased from Macklin Inc.

## 2 METHODS

Healthy Sprague Dawley (SD) rats were randomly divided into the following five groups: control group (no UVA irradiation), model group (UVA irradiation + solvent application), Acacetin low dose group (UVA irradiation +40 mg/kg/d Acacetin), Acacetin high dose group (UVA irradiation +80 mg/kg/d Acacetin) and Vitamin E (VE) group (UVA irradiation +500 mg/kg/d VE). For UVA irradiation, straight tube reflective UVA lamps (Huaqiang, China; UV wavelength range between 320–400 nm) were used. All rats received shaving of the back, and each group of rats was coated with the corresponding concentration of the solvent. A distance of 20 cm was kept between the back of rats and the lamp source in order to maintain the required radiation dose (300 mJ/cm^2^) for 1 h on alternate days (irradiation started 1 h after administration) for 1 month.

HDF cells were incubated in dulbecco's modified eagle medium containing 1% antibiotics and 10% foetal bovine serum at 37°C. An in vitro acute photoaging cell model was constructed using UVA irradiation of HDF at 20 J/cm^2^. In the experiment, cells were divide cells were divided into the following five groups: control group (without UVA irradiation and Acacetin treatment), UVA group (only UVA irradiation), 5‐Ac group (UVA irradiation plus 5 μg/mL Acacetin), 10‐Ac group (UVA irradiation plus 10 μg/mL Acacetin), 20‐Ac group (UVA irradiation plus 20 μg/mL Acacetin). After 24 h of cell plating, cells were co‐incubated with different concentrations of Acacetin (5, 10, 20 μg/mL) for 12 h, and then subjected to UVA irradiation (irradiation dose of 20 J/cm^2^).^9^ In second experiments, cells were divided into the following five groups: control group (without UVA irradiation and Acacetin treatment), UVA group (only UVA irradiation), Acacetin group (UVA irradiation plus 20 μg/mL Acacetin), 3‐TYP group (UVA irradiation plus 50 mM 3‐TYP), Acacetin +3‐TYP group (UVA irradiation plus 20 μg/mL Acacetin and 50 mM 3‐TYP). Additional details of relevant materials and methods are provided as supplementary information (see Appendix S1).

## 3 RESULTS

### 3.1 Acacetin alleviates skin damage and oxidative stress in UVA‐irradiated rat skin

**FIGURE 1:**
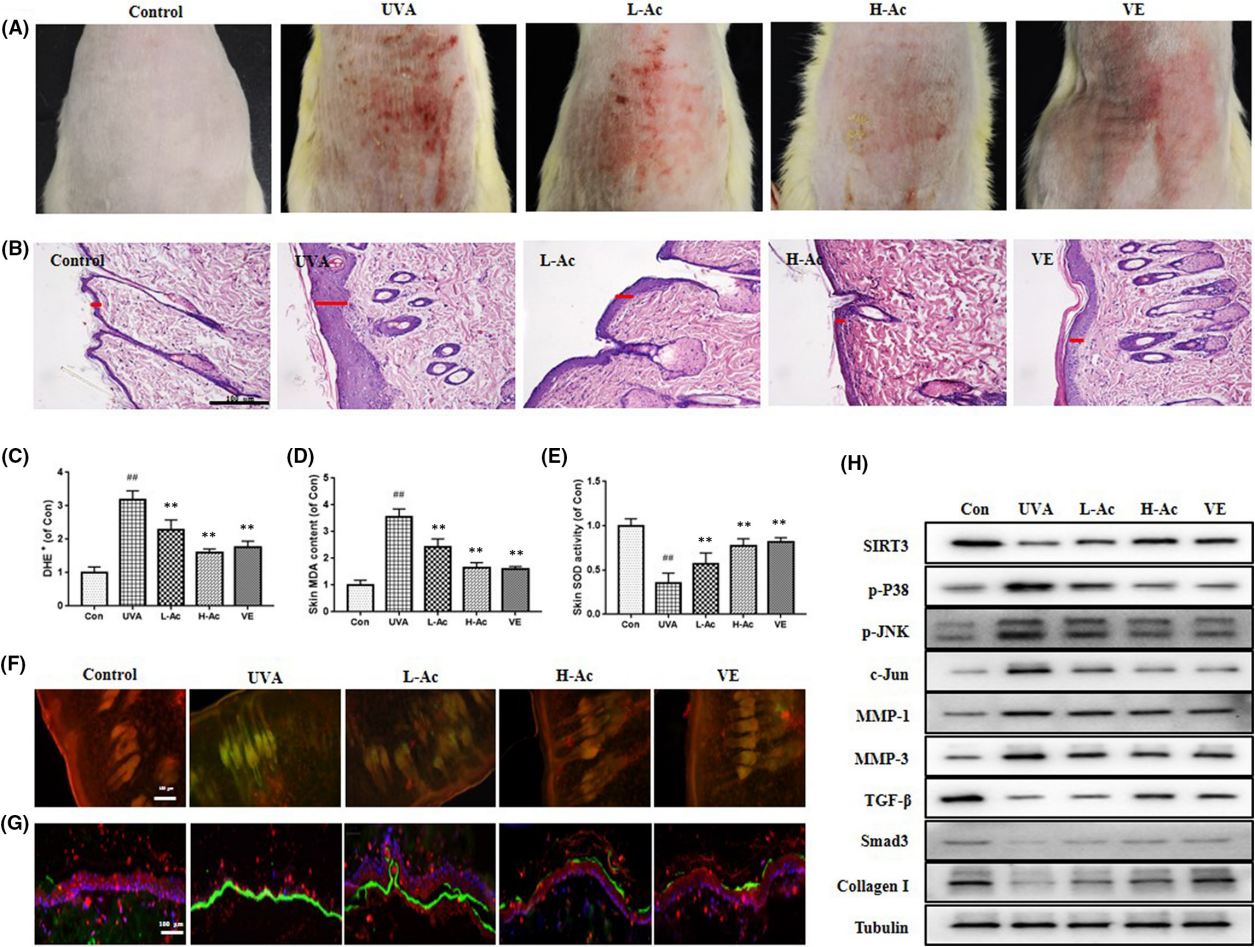
Acacetin alleviates skin damage and oxidative stress in UVA‐irradiated rat skin. (A) Macroscopic changes in the dorsal skin of rats (*n* = 6). (B) Representative haematoxylin and eosin‐stained sections and thickness of epidermis, and the red line indicates keratinisation of the epidermis (*n* = 3). (C) The relative fluorescence intensity of dihydroethidium (DHE^+^) in each group. The contents of MDA (D) and SOD (E) of skin in each group (*n* = 6). (F) Acacetin partially restores the decrease in mitochondrial membrane potential induced by UVA irradiation (*n* = 3). (G) Representative immunofluorescence staining for Collagen I (red) and MMP‐1 (green) (*n* = 3). (H) The expressions of SIRT3, p‐P38 MAPK, p‐JNK, MMP‐1, MMP‐3, TGF‐β, TβRII, Smad3, Collagen I in each group (*n* = 3). VE: vitamin E; L‐Ac: 40 mg/kg/d Acacetin; H‐Ac: 80 mg/kg/d Acacetin. Data represent as means ± SD. In (C–E), #*p* < 0.05 compared with the control group and *p < 0.05 compared with the UVA group

The bare skin of rats in the UVA group showed significant signs of erythema and wrinkles after UVA radiation compared with the control group, whereas the erythema and wrinkles were less pronounced in the groups coated with Acacetin than in the UVA group, and application of Acacetin reduced the increase in epidermis caused by UVA (Figure 1 A). As shown in Figure 1 B, examination of the epidermal thickness of the rats by HE staining showed that the epidermal thickness of the UVA group was much greater than that of the control group, while the epidermal increase induced by UVA was significantly reduced by Acacetin application. In Figure 1C–E, UVA irradiation increased the levels of ROS and MDA and decrease the level of SOD in the UVA group compared with the control group. However, in the case of Acacetin application, the levels of ROS and MDA was decreased and the level of SOD was increased, indicating that Acacetin can reduce the level of oxidative stress. In Figure 1F, UVA irradiation significantly reduced the mitochondrial membrane potential (red/green ratio), which was significantly restored by the application of Acacetin.

**FIGURE 2:**
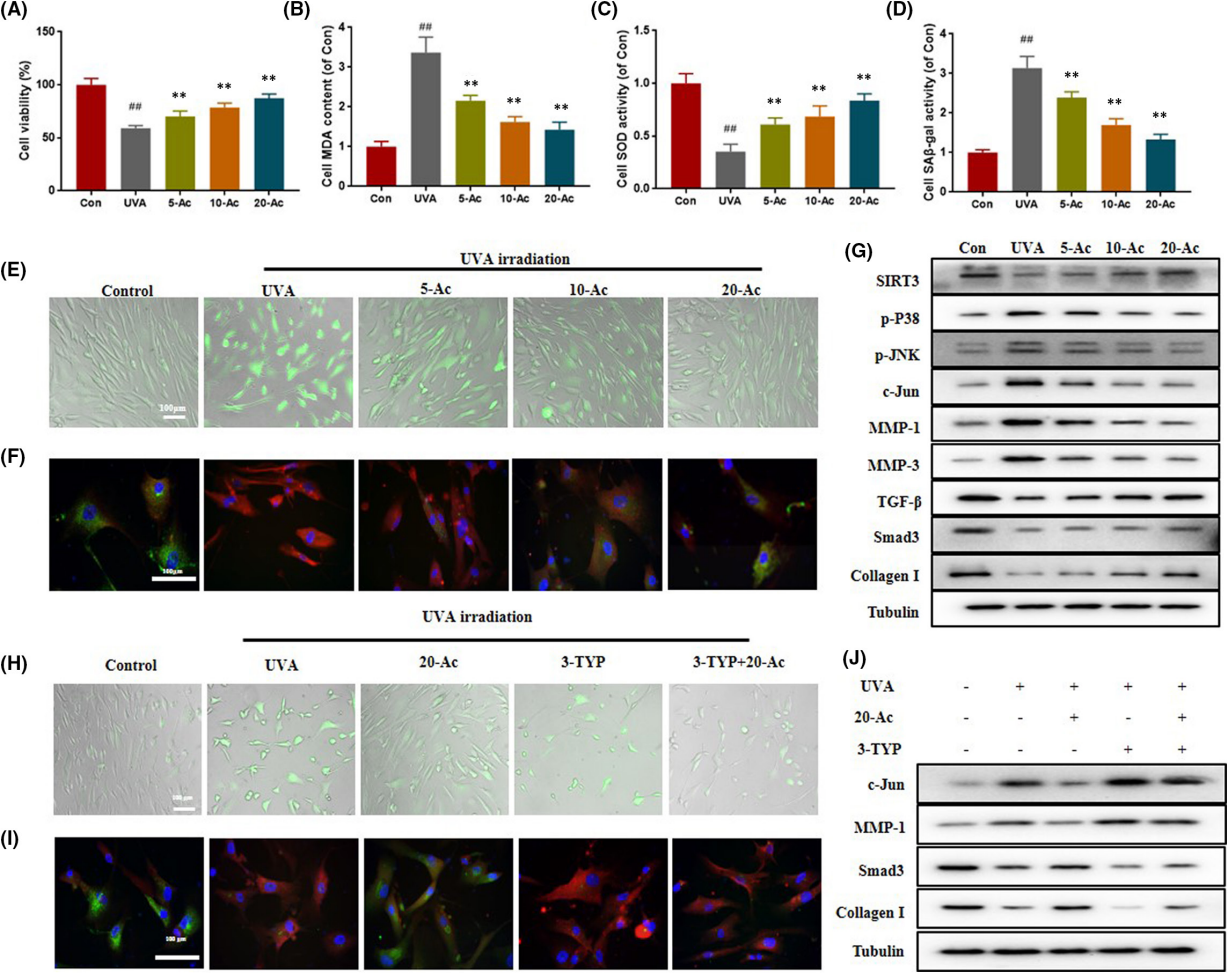
Acacetin reduces oxidative stress levels and regulates related proteins' expressions of HDF under UVA irradiation. (A) Acacetin improves the cell viability of HDF under UVA‐irradiation. The contents of MDA (B), SOD (C) and SA‐β‐gal (D) in different group. (E) Representative images of DCF^+^ probe staining in HDF under UVA irradiation. (F) Representative images of immunofluorescence staining for Collagen I (green) and MMP‐1 (red). (G) Acacetin regulates the expressions of SIRT3, p‐P38 MAPK, p‐JNK, MMP‐1, MMP‐3, TGF‐β, TβRII, Smad3 and Collagen I. (H) Representative images of DCF^+^ probe staining in HDF under UVA irradiation. (I) Representative images of immunofluorescence staining for Collagen I (green) and MMP‐1 (red). (J) Acacetin regulates the protein expression levels of c‐Jun, MMP‐1, Smad3 and Collagen I. 5‐Ac: 5 μg/ml Acacetin; 10‐Ac: 10 μg/ml Acacetin; 20‐Ac: 20 μg/ml Acacetin. Data represent as means ± SD. In (A–D), #*p* < 0.05 compared with the control group. *p < 0.05 compared with the UVA group

In Figure 2G, UVA exposure significantly increased the expression of MMP‐1 (green) while inhibiting the expression of Collagen I (red) compared with the control group. Also, UVA exposure increased the expressions of p‐P38, p‐JNK, c‐Jun, MMP‐1 and MMP‐3, while inhibited the expressions of SIRT3, TGF‐β, Smad3 and Collagen I (Figure 1H and Figure S1). Acacetin administration reversed this trend.

### 3.2 Acacetin reduces oxidative stress levels and regulates related proteins' expressions of HDF under UVA irradiation

As shown in Figure 2A, UVA irradiation induced a significant decrease in cell viability, whereas the treatment of Acacetin alleviated UVA irradiation induced death in a dose‐dependent manner. UVA irradiation greatly increase the production of MDA, senescence‐associated β‐Galactosidase (SA‐β‐gal) and ROS and decreased the activity of SOD in HDF. Interestingly, Acacetin treatment significantly inhibited the levels of MDA, SA‐β‐gal and ROS, which in turn increased the level of SOD (Figure 2B‐E). Results of immunofluorescence analysis showed that MMP‐1 (red) was significantly increased and collagen I (green) was significantly decreased in the UVA group compared with the control group, and this was reversed in the Acacetin‐treated groups (Figure 2F). In in vitro experiments, Acacetin addition also reduced the UVA‐induced increase in p‐P38, p‐JNK, c‐Jun, MMP‐1 and MMP‐3 and increased the expression of SIRT3, TGF‐β, Smad3 and Collagen I (Figure 2G and Figure S2).

The protective effect of Acacetin on cell exposure to UVA irradiation was further investigated using 3‐TYP, an inhibitor of SIRT3. As shown in Figure 2H, the ROS level of the UVA + 20‐Ac + 3‐TYP was significantly higher than that in the UVA + 20‐Ac group. The original increases in c‐Jun and MMP‐1 and decreases in Collagen I and Smad3 stimulated by UVA irradiation were decreased and increased by Acacetin, respectively, and this trend was blocked by the addition of 3‐TYP (Figure 2I, J and Figure S3).

## 4 DISCUSSION

UVA exposure eventually led to depletion of cellular antioxidant levels, reduced SOD activity and increased MDA and ROS levels in skin tissue of rat and HDF. However, application of Acacetin to the back of rats resulted in a decrease of oxidative stress levels. Similarly, in UVB‐induced photoaging in HDF and HaCaT cells, Acacetin regulates MMP‐1 by modulating the MAPK signalling pathway.^10^

Several natural substances have been reported to prevent UV‐induced aging through their antioxidant properties. The protective effect of topical application of orange peel cold‐pressed oil (a rich polymethoxyflavones source) against UV‐induced skin damage contributes to the maintenance of dermal structure and enhances superoxide dismutase, catalase and glutathione peroxidase in skin tissues; the protective effect of isoflavone extracts from soybean cake against UVB‐induced skin photoaging by inhibiting apoptosis and inflammatory responses. We sought to explore the molecular protective mechanism of Acacetin against UVA‐induced photoaging and results suggested that Acacetin treatment significantly could restore the level of SIRT3, reduce the accumulation of ROS, inhibit the expression of p38MAPK/AP‐1/MMPs and promote the TGF‐β/Smad3 pathway, thereby reducing collagen decay in a UVA irradiation model.

It has been previously reported that UVA activates MAPK via ROS, triggering the activation of AP‐1 and c‐Fos and causing skin inflammation.^11^ We observed that Acacetin treatment inhibited the increase of p‐P38, p‐JNK and c‐Jun induced by UVA irradiation, similar to previous study, with the difference that Acacetin did not alter p38 phosphorylation in UVB‐induced photoaging.^10^ Protein phosphorylation is one of the major mechanisms mediating transcription factor activity, and phosphorylated c‐Jun heterodimerizes with phosphorylated c‐Fos to form the highly active AP‐1 complex, which is essential for transcription of MMP‐1.^12^

The degradation of collagen is mainly regulated by MMPs, which play an important role in UVA‐induced photoaging.^13^ To elucidate the mechanism of Acacetin on UVA‐induced photoaging, we investigated the expression of MMP‐1 and MMP‐3 in skin and cells. The results showed that treatment with Acacetin prior to UVA irradiation significantly reduced the expression of MMP‐1 and MMP‐3. Elevated ROS impairs transforming growth factor‐β (TGF‐β) by reducing Smad3 protein levels, which results in reduced expression of connective tissue type I collagen.^14^ The use of Acacetin before UVA irradiation increased TGF‐β and Smad3. Taken together, this could explain the increased expression of collagen in the skin or cells.

Decreased level of SIRT3 has been observed in tissue and cellular responses to UVA radiation. SIRT3 is known to play an important role in regulating mitochondrial functions, including energy metabolism and ROS homeostasis.^15^ UVA photoaging is mediated by oxidative stress. By co‐treatment of cells with 3‐TYP (inhibitor of SIRT3) and Acacetin, the lowering effect of Acacetin on ROS and MMP‐1 and the enhancing effect on Collagen I were lost, further suggesting that the protective effect of Acacetin against UVA photoaging is mediated by SIRT3 action.

This study showed that Acacetin prevents UVA‐induced photoaging by decreasing oxidative stress levels and increasing SIRT3 expression. In addition, Acacetin effectively alleviated the photoaging process by inhibiting UVA‐induced MMPs expressions and suppressing the increase of pro‐inflammatory factors, promoting TGF‐β and Smad3. Therefore, Acacetin can be used as a potential natural sunscreen ingredient.

## AUTHOR CONTRIBUTIONS

**Jing Mu:** Conceptualization (lead); funding acquisition (lead); writing – review and editing (equal). **Hong Chen:** Data curation (equal); methodology (equal); writing – original draft (equal); writing – review and editing (equal). **Mengyi Ye:** Methodology (equal); software (equal); writing – original draft (equal); writing – review and editing (equal). **Xiaoxia Zhang:** Formal analysis (equal); methodology (equal); writing – original draft (equal); writing – review and editing (equal). **Huisheng Ma:** Conceptualization (supporting); funding acquisition (supporting); writing – review and editing (equal).

## CONFLICT OF INTEREST

The authors declare that they have no competing interests.

## Supporting information


Appendix S1

Figure S1–S3

Data S1

## Data Availability

Data available on request from the authors
