# Supplementary material for: Acacetin resists UVA photoaging by mediating the SIRT3/ROS/MAPKs pathway
Source: J Cell Mol Med. 2022 Jun 28;26(16):4624–8. doi: 10.1111/jcmm.17415 (PMC9357640; doi:10.1111/jcmm.17415)
Supplement: Supplementary file 1 — Appendix S1 [file JCMM-26-4624-s001.docx]

**Supporting Information**

1. **Materials and Methods**

**2.1 Reagents and antibodies**

Acacetin (A860582, purity ≥ 98%) was purchased from Macklin Inc. (Beijing, China). Anti-Sirtuin-3 (SIRT3, 2627), Anti-mitogen-activated protein kinases (MAPK p38, 8690), Anti-p-JNK (9255), Anti-c-Jun (9165), Anti-MMP-1 (54376), Anti-MMP-3 (14351), Anti-TGF-β (3711), Anti-Smad3 (9523) and Anti-rabbit IgG (7074) antibodies were purchased from Cell Signaling Technology, Inc. (US) and Collagen I (AF7001, Affinity Biosciences, China).

**2.2 Animals and photoaging model establishment**

Healthy adult SD rats (180-220 g) were purchased Jiangsu ALF Biotechnology Co., LTD. After 1 week of adaptation, the rats were randomly divided into five groups: control group (no UVA irradiation), model group (UVA irradiation + solvent application), Acacetin low dose group (UVA irradiation + 40 mg/kg/d Acacetin), Acacetin high dose group (UVA irradiation + 80 mg/kg/d Acacetin) and Vitamin E (VE) group (UVA irradiation + 500 mg/kg/d VE). The hair was removed from the neck to the rump of the rats so that their backs were completely exposed to UV irradiation. For UVA irradiation, straight tube reflective UVA lamps (Huaqiang, China; UV wavelength range between 320-400 nm) were used. Rats were irradiated on the back for 1 h on alternate days (irradiation started 1 h after administration) for one month. After successful modeling, rats were sacrificed, blood from the abdominal aorta was collected, and serum was collected by centrifugation, and skin tissues from the irradiated area were collected. All samples were stored at -20℃ for use. The composition of the solvent is 30% ethanol plus 70% propylene glycol.

**2.3 Biochemical indicators**

The levels of malondialdehyde (MDA) and superoxide dismutase (SOD) activity were measured by commercial MDA (A003-1-2, Jiancheng, China) and SOD (A001-1-2, Jiancheng, China) kits according to manufacturers’ instructions.

SA-β-gal activity was measured 72 h after UVA radiation using the cell senescence assay kit (M013, Jiancheng, China) according to the manufacturer’s instructions.

**2.4 Mitochondrial membrane potential detection**

ΔΨm, the mitochondrial membrane potential, has been used as an indicator of cell health, and changes in mitochondrial membrane potential can be detected using JC-1 [^13^](#_ENREF_13). In healthy cells with high mitochondrial membrane potential, JC-1 forms complexes called J-aggregates (red), while in cells with low mitochondrial membrane potential, JC-1 remains in monomeric form (green). The membrane potential assay was performed using the Mitochondrial Membrane Potential Assay Kit JC-1 (M8650, Solarbio, China), following the manufacturer’s protocol.

**2.5 Histopathology detection**

Skin samples from the back were collected, fixed in 4% paraformaldehyde and embedded in paraffin for hematoxylin and eosin or immunohistochemical staining as previously described [^14^](#_ENREF_14). For HE staining, sections were stained with hematoxylin solution for 1 min, then stained with eosin solution for 1 min, dehydrated with graded alcohol and clarified in xylene. For immunohistochemistry, sections were incubated with anti-Collagen I antibody (AF7001, Affinity Biosciences, China) or anti-MMP-1 antibody (ab52631, abcam, UK) overnight at 4°C, followed by incubation with the secondary antibody Goat Anti-Rabbit IgG (Alexa Fluor 594, ab150080, abcam, UK) or Goat Anti-Mouse IgG H&L (Alexa Fluor 488, ab150117, abcam, UK) followed by color development with diaminobenzidine.

**2.6 Cell culture and modeling**

An *in vitro* acute photoaging cell model was constructed using UVA irradiation of HDF at 20 J/cm^2^ ccroding to a previous study [^15^](#_ENREF_15). In the experiment, cells were divided into five groups: control group (without UVA irradiation and Acacetin treatment), UVA group (only UVA irradiation), 5-Ac group (UVA irradiation plus 5 μg/mL Acacetin), 10-Ac group (UVA irradiation plus 10 μg/mL Acacetin), 20-Ac group (UVA irradiation plus 20 μg/mL Acacetin). After 24 h of cell plating, cells were co-incubated with different concentrations of Acacetin (5, 10, 20 μg/mL) for 12 h, and then subjected to UVA irradiation (irradiation dose of 20 J/cm^2^).

In the drug mechanism experiments, cells were divided into five groups: control group (without UVA irradiation and Acacetin treatment), UVA group (only UVA irradiation), Acacetin group (UVA irradiation plus 20 μg/mL Acacetin), 3-TYP group (UVA irradiation plus 50 mM 3-TYP), Acacetin + 3-TYP group (UVA irradiation plus 20 μg/mL Acacetin and 50 mM 3-TYP).

**2.7 Detection of cell viability**

The viability of HDF was detected by MTT kit (C0009S, Beyotime, China). Briefly, cells in log phase were collected and placed into 96-well plates and incubated with drugs for 24 h. 10 µL of MTT solution per well and incubation was continued for 4 h. Then, 100 µL of Formazan solution was added to each well, properly mixed and incubated at 37℃ for 3 h, and absorbance was measured at 570 nm.

**2.8 Detection of ROS**

Flow cytometry was used to determinate the intracellular ROS. The cultured HDF (4 × 10^5^ cells) were seeded into 6-well plates, and then treated according to modeling method. After successful modelling of the cellular UVA damage model, the cells were incubated with 20, 70-dichlorodihydrofluorescein-diacetate (DCF-DA, S0033M, Beyotime, China) solution for 30 min, and then immediately assayed on the machine.

**2.9 Western blot**

Total protein samples were obtained from UVA-irradiated skin tissues, electrophoresed on 10% SDS-PAGE gels and then transferred to polyvinylidene fluoride (PVDF) membranes. After sealing with 5% BSA for 1 h, the membranes were incubated with primary antibodies at 4°C overnight, followed by incubating with corresponding HRP-coupled secondary antibodies for 1 h. Finally, the signals were detected using the ECL method and analyzed by Image J software.

**2.10 Statistical analysis**

Mean comparisons were performed using a two-group Student’s t-test and a multi-group one-way analysis of variance (ANOVA). All data were expressed as mean ± standard deviation and analyzed by GraphPad Prism 8.0. values of p < 0.05 were considered statistically significant.
