# Supplementary material for: Acacetin resists UVA photoaging by mediating the SIRT3/ROS/MAPKs pathway
Source: J Cell Mol Med. 2022 Jun 28;26(16):4624–8. doi: 10.1111/jcmm.17415 (PMC9357640; doi:10.1111/jcmm.17415)

Fig. S1 Protein quantification of SIRT3, p-P38 MAPK, p-JNK, MMP-1, MMP-3, TGF-β, TβRII, Smad3, Collagen I in UVA-irradiated rat skin.

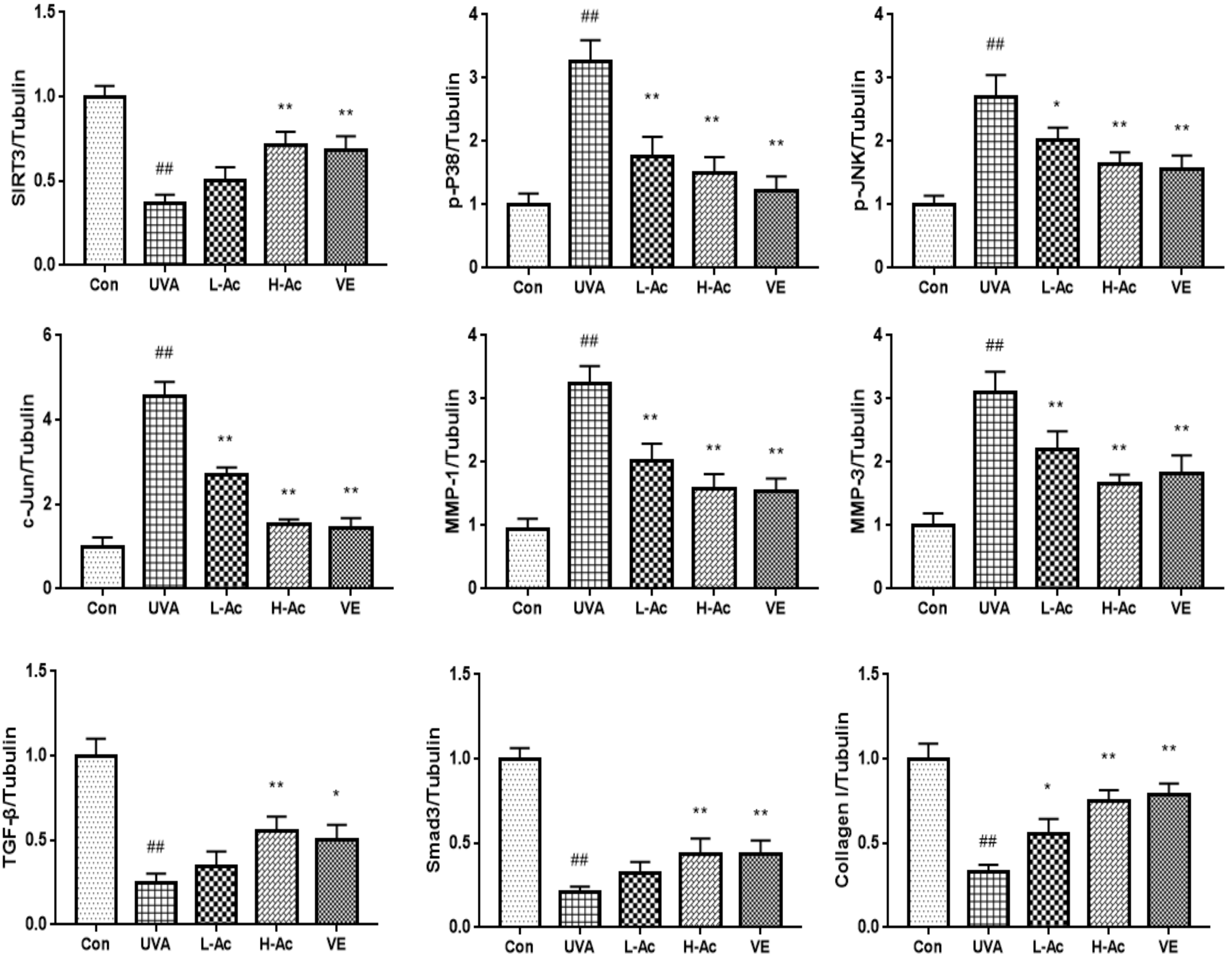

Fig. S2 Protein quantification of SIRT3, p-P38 MAPK, p-JNK, MMP-1, MMP-3, TGF-β, TβRII, Smad3, Collagen I in UVA-irradiated HDF.

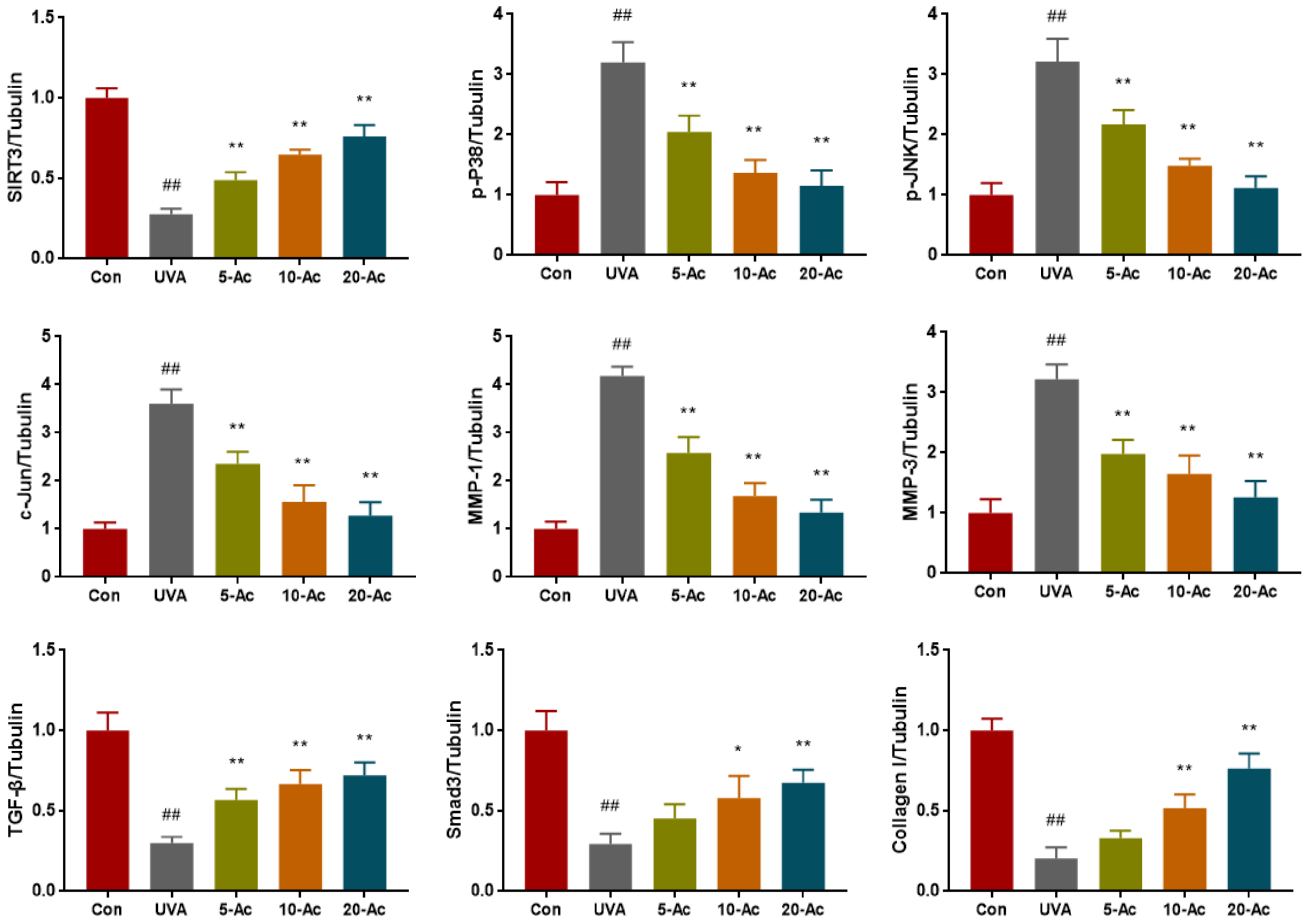

Fig. S3 Protein quantification of c-Jun, MMP-1, Smad3 and Collagen I in UVA-irradiated HDF.

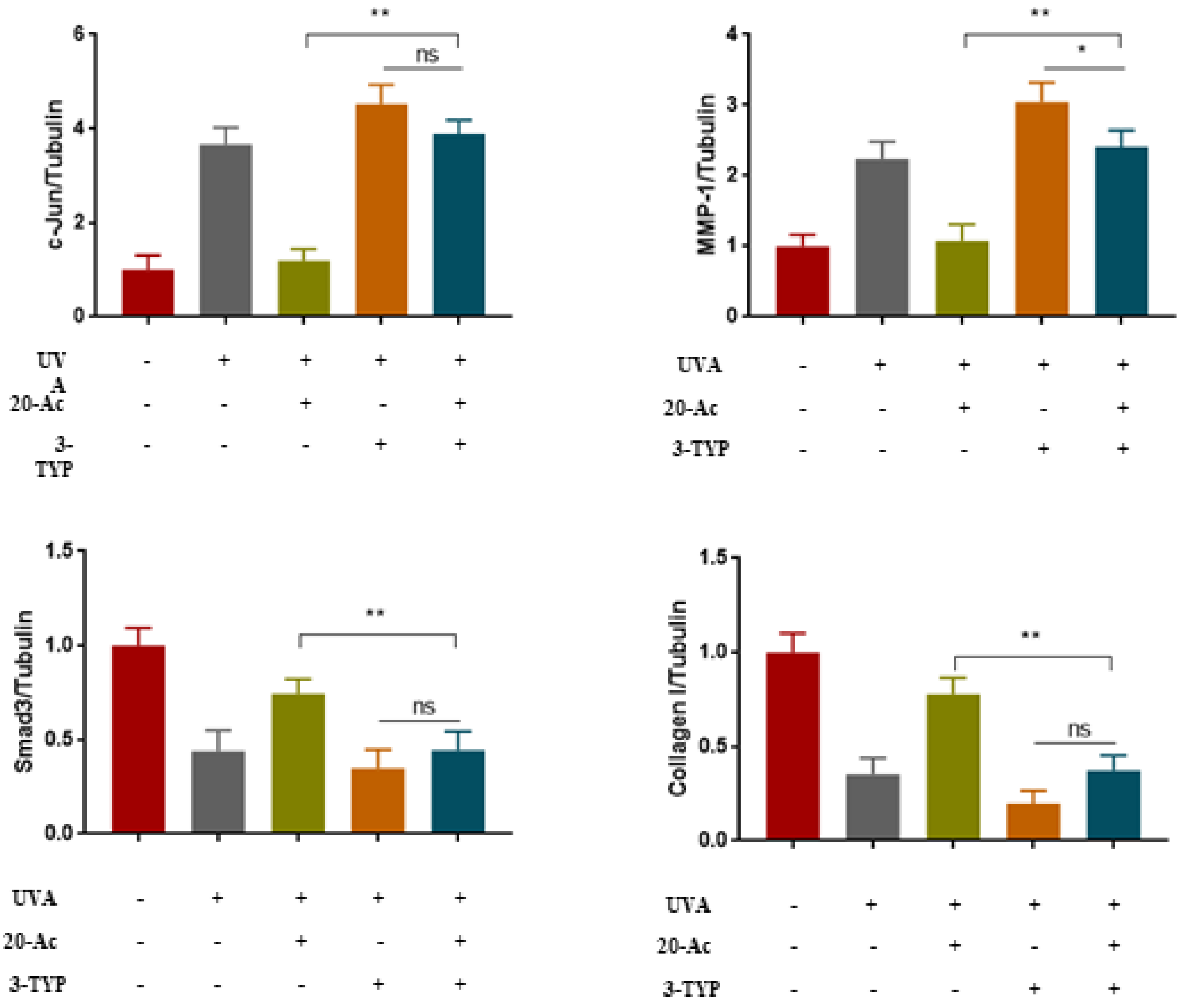

Supplement: Supplementary file 2 — Figure S1–S3 [file JCMM-26-4624-s002.pdf]
