# Supplementary material for: Acacetin resists UVA photoaging by mediating the SIRT3/ROS/MAPKs pathway
Source: J Cell Mol Med. 2022 Jun 28;26(16):4624–8. doi: 10.1111/jcmm.17415 (PMC9357640; doi:10.1111/jcmm.17415)
Supplement: Supplementary file 3 — Data S1 [file JCMM-26-4624-s003.ppt]

## Slide 1
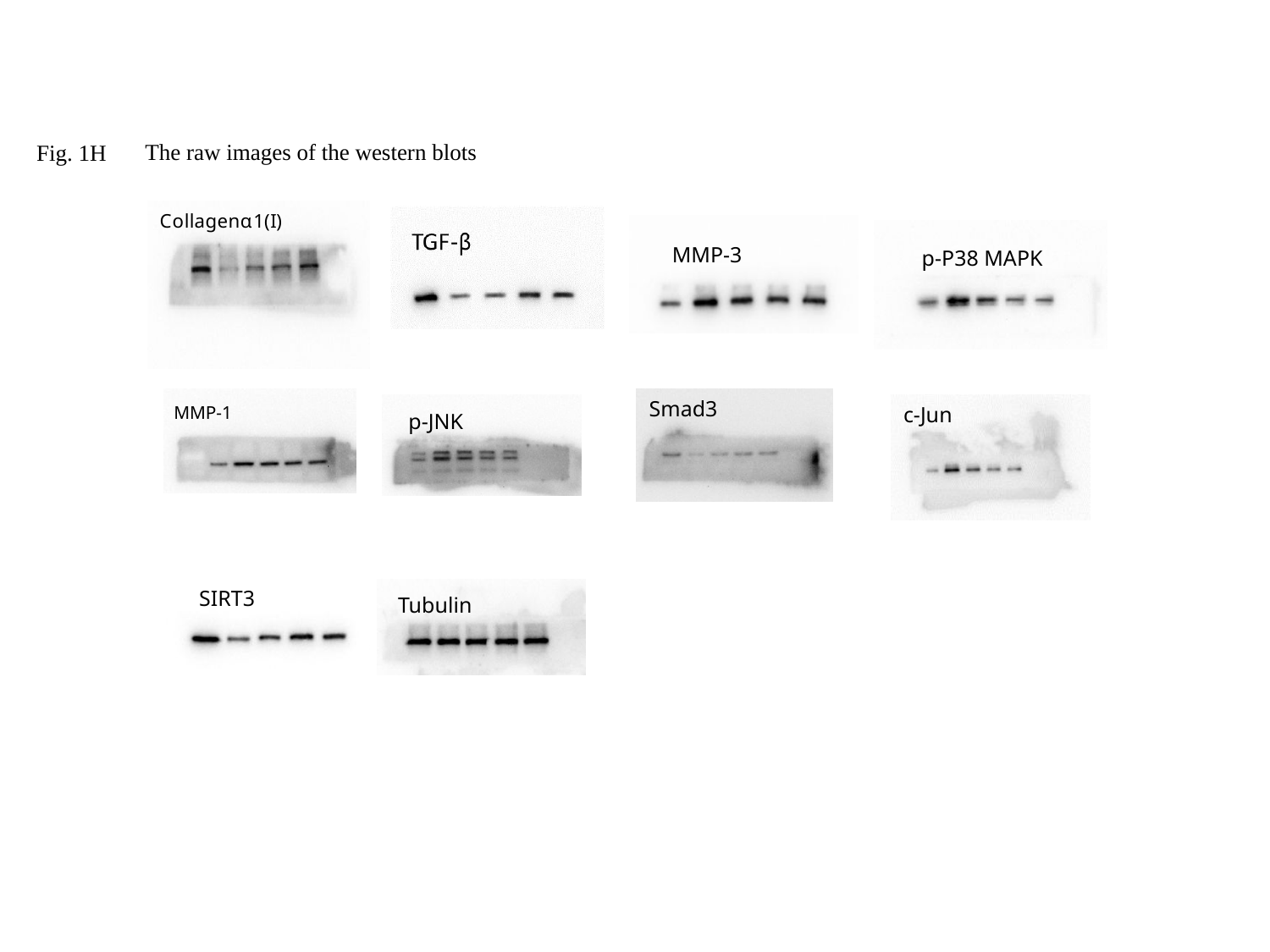

The raw images of the western blots
Fig. 1H
MMP-3
p-P38 MAPK
MMP-1
Smad3
c-Jun
p-JNK
SIRT3
Tubulin

## Slide 2
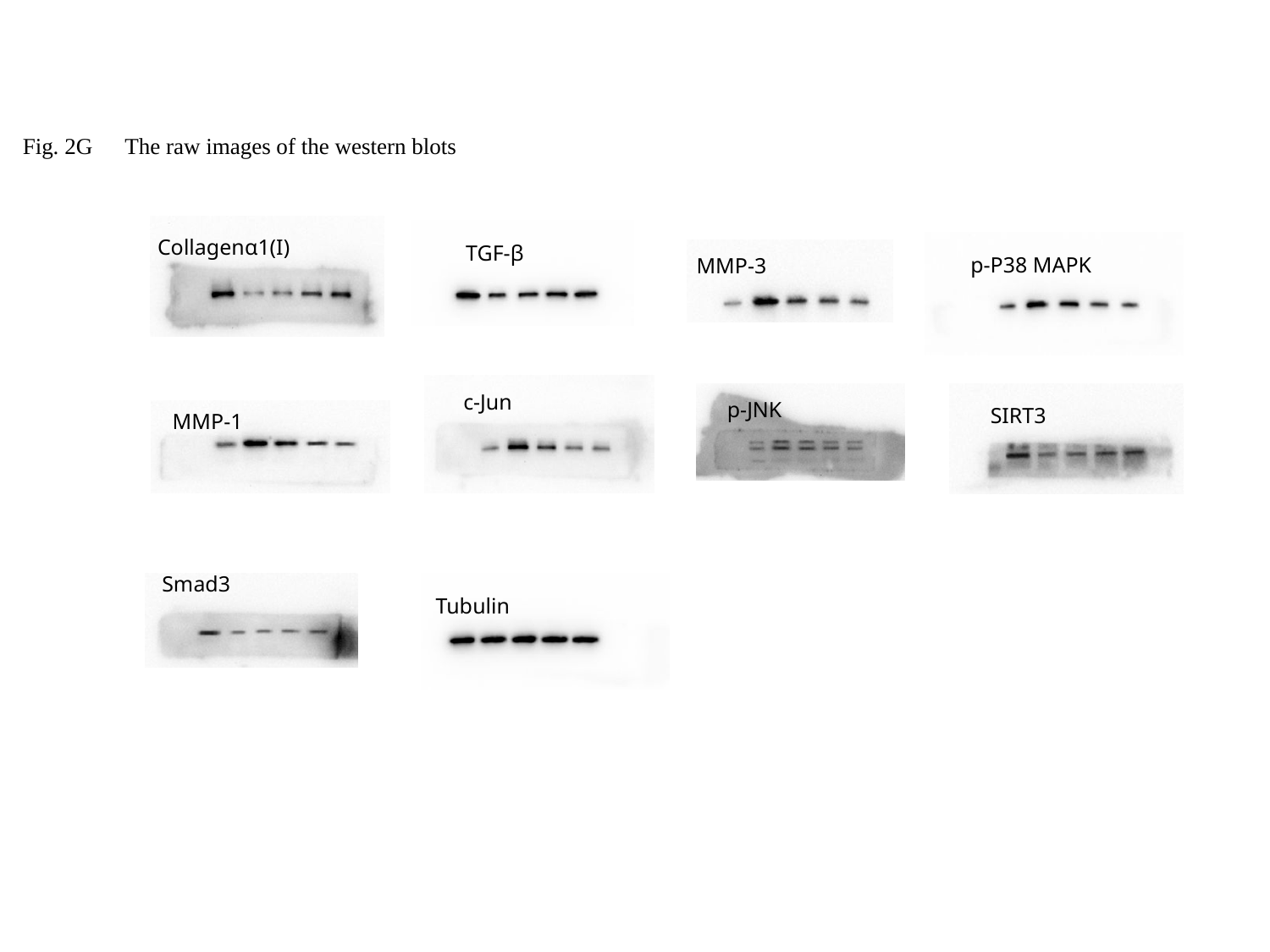

Fig. 2G
The raw images of the western blots
Collagenα1(I)
TGF-β
p-P38 MAPK
MMP-3
c-Jun
p-JNK
SIRT3
MMP-1
Smad3
Tubulin

## Slide 3
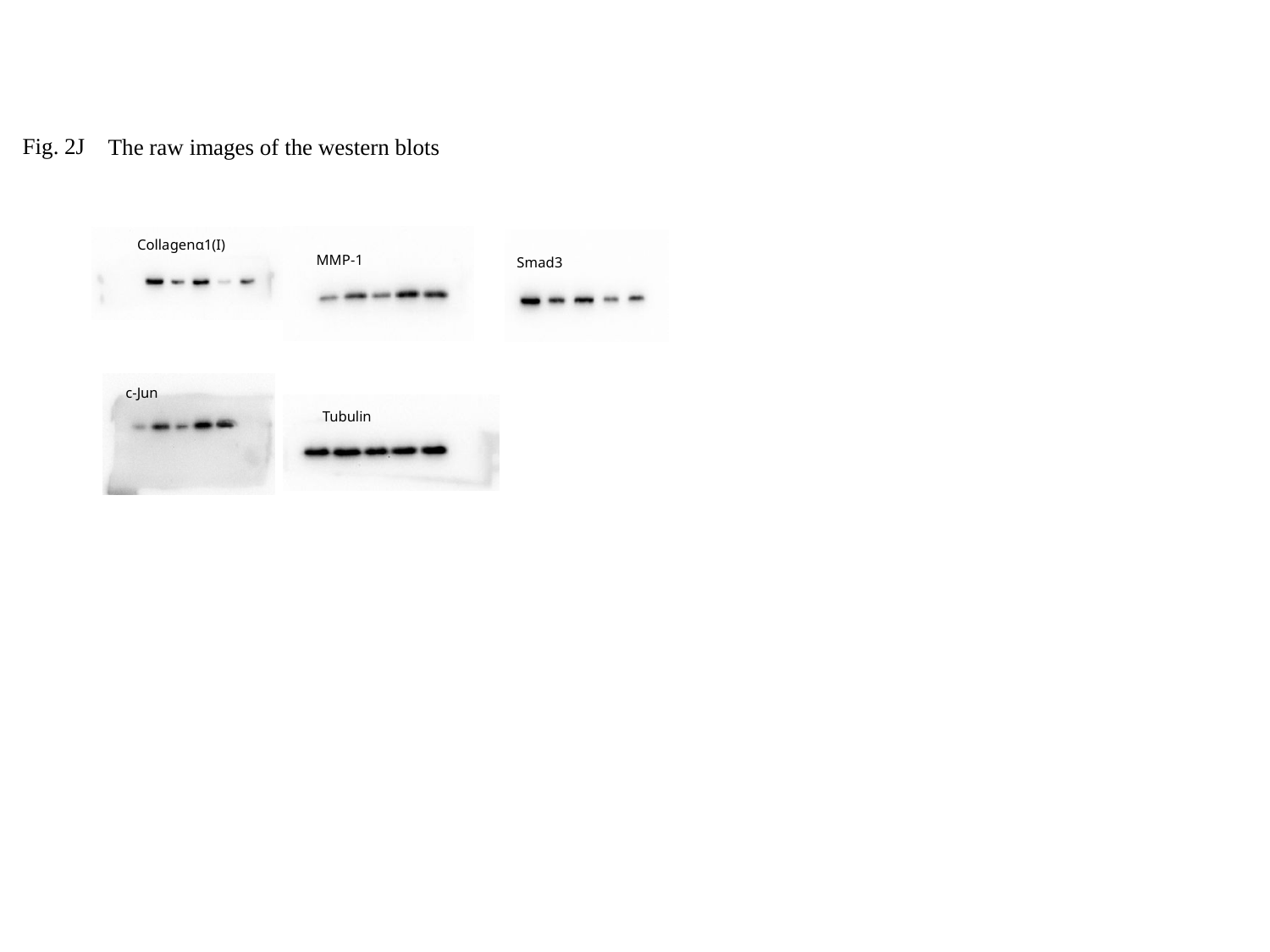

Fig. 2J
The raw images of the western blots
MMP-1
Collagenα1(I)
Smad3
c-Jun
Tubulin
